# Reply to Nduka et al. Comment on “Kehrer et al. Using High-Resolution Ultrasound to Assess Post-Facial Paralysis Synkinesis—Machine Settings and Technical Aspects for Facial Surgeons. *Diagnostics* 2022, *12*, 1650”

**DOI:** 10.3390/diagnostics12102432

**Published:** 2022-10-08

**Authors:** Andreas Kehrer, Lukas Prantl, Samuel Knoedler, Leonard Knoedler

**Affiliations:** Department of Plastic, Hand and Reconstructive Surgery, University Hospital Regensburg, 93053 Regensburg, Germany

We thank Dr. Nduka et al. for this interesting article [1]. We very much enjoyed reading the detailed Instructions for Sonography of the Mimic Musculature [2]. We found the schematic, yet practice-orientated drawings helpful for visualizing the complex facial anatomical relationships and different planes. We see our structured working protocol as add-on to their work [3].

Together with other advancements in clinical facial palsy (FP) diagnostics and basic scientific translational efforts, such as automated grading systems or axon quantification, high-resolution ultrasound (HRUS) enlarges the FP surgeon’s arsenal and understanding of FP disease [4,5,6,7,8,9].

Reading both publications, FP surgeons with basic levels of knowledge of facial HRUS may enhance their clinical workflow and refine their diagnostic setting in FP patients. Such an evidence-based approach might promote FP patient care, scientific data acquisition, and cost-effectiveness.

In future studies, the combination of facial HRUS and machine learning should explore the feasibility of automatizing this diagnostic step. Overall, facial HRUS represents a promising research field for both basic and clinical studies, and we are looking forward to future developments in facial HRUS.
